# Biochar co-compost increases the productivity of *Brassica napus* by improving antioxidant activities and soil health and reducing lead uptake

**DOI:** 10.3389/fpls.2024.1475510

**Published:** 2024-11-12

**Authors:** Wenjie Jiang, Ying Liu, Jing Zhou, Haiying Tang, Guiyuan Meng, Xianrui Tang, Yulong Ma, Tuyue Yi, Fahmy Gad Elsaid

**Affiliations:** ^1^ School of Agriculture and Biotechnology, Hunan University of Humanities, Science and Technology, Loudi, China; ^2^ Shuangfeng Agriculture and Rural Bureau, Loudi, China; ^3^ Department of Biology, College of Science, King Khalid University, Abha, Saudi Arabia

**Keywords:** antioxidants, health risks, lead stress, oil contents, phytostabilization

## Abstract

Lead (Pb) is a serious toxic metal without any beneficial role in the biological system. Biochar (BC) has emerged as an excellent soil amendment to mitigate Pb toxicity. The impact of BC co-compost (BCC) in mitigating the toxic impacts of Pb has not been studied yet. Therefore, this study aimed to evaluate the potential of BC and BCC in improving the growth, physiological, and biochemical traits of *Brassica napus* and soil properties and reducing health risks (HR). The study was comprised of different Pb concentrations (control and 100 mg kg^-1^) and organic amendments (control, BC, compost, and BCC). The results indicated that Pb stress reduced the growth, photosynthetic pigments, seed yield, and oil contents by increasing hydrogen peroxide (H_2_O_2_) production and Pb uptake and accumulation in plant tissues and decreasing photosynthetic pigment and nutrient availability. The application of BCC alleviated the adverse impacts of Pb and improved seed production (40.24%) and oil yield (11.06%) by increasing chlorophyll a (43.18%) and chlorophyll b (25.58%) synthesis, relative water content (23.89%), total soluble protein (TSP: 23.14%), free amino acids (FAA: 26.47%), proline (30.98%), APX (40.90%), CAT (32.79%), POD (24.93%), and SOD (33.30%) activity. Biochar co-compost-mediated increase in seed and oil yield was also linked with a reduced accumulation of Pb in plant parts and soil Pb availability and improved the soil-available phosphorus, potassium, total nitrogen, soil organic carbon (SOC), and microbial biomass carbon (MBC). Furthermore, BCC also reduced the bioaccumulation concentration, daily metal intake, hazard index, and target hazard quotient. In conclusion, application of BCC can increase the growth, yield, and oil contents of *Brassica napus* by improving the physiological and biochemical traits and soil properties and reducing the Pb uptake.

## Introduction

Soil contamination with heavy metals (HMs) is a serious concern globally due to their toxic impacts on humans, plants, and the environment (Paini et al., 2016). Heavy metals inhibit plant growth and productivity and pose serious health issues (Tovar-Sánchez et al., 2018). Lead (Pb) is one of the most toxic metals that can cause adverse impacts on humans, particularly in children and pregnant women (WHO, 2019). It has wide uses in batteries, pigments, ammunition, cable sheathing, crystal glass, weights, and radiation protection. Therefore, it has the potential to contaminate environment and agricultural soils (Entwistle et al., 2019). Crops grown on Pb-polluted soils are a major reason of Pb entry into the human food chain (WHO, 2019). Thus, serious measures should be adopted to address this problem (Egendorf et al., 2020).

Lead is a dangerous metal, and it negatively affects seed germination and growth and the yield of crops (Yahaghi et al., 2019; Yadav et al., 2023). Lead seriously reduces root growth and nutrient and water uptake, resulting in profound growth losses (Chen et al., 2023). It also damages the photosynthetic apparatus and reduces chlorophyll synthesis and photosynthetic efficiency, leading to a reduction in assimilate production and plant biomass production (Emamverdian et al., 2015; Karumannil et al., 2023; Sorce et al., 2023). Lead toxicity also leads to the excessive production of reactive oxygen species (ROS) that causes oxidation of proteins, lipids, and DNA (Collin et al., 2022; Wakabayashi et al., 2023). Plants use different strategies, including antioxidant defense system, osmolyte accumulation, and restricted Pb uptake, to counter the toxic impacts of Pb (Garcia and Palacio, 2020; Sytar et al., 2021). These strategies adopted by plants are not enough to mitigate the adverse impacts of Pb; thus, exogenous techniques can provide the solution to combat the toxic effects of Pb.

Different physical, chemical, and biological strategies are being used for the remediation of polluted soils (Irshad et al., 2021). Physical and chemical techniques are time-consuming, labor-intensive, and energy-consuming, and they also produce toxic wastes (Sabir et al., 2020). In this context, application of organic amendment (OA) can offer a potential solution to reduce the toxic effects of HMs (Hu et al., 2020). In recent years, biochar (BC) showed great results to restore HM-polluted soils owing to its recalcitrant nature, cost-effectiveness, and environment-friendly benefits (Kamran et al., 2019a). Biochar is a carbon-rich product produced as a result of pyrolysis (Abbas et al., 2020), and it has unique properties including a porous structure and a higher surface area. These features make BC an important amendment to improve soil fertility, carbon sequestration, and metal stabilization (Hu et al., 2020). Biochar improves soil organic carbon (SOC), nutrient availability, and microbial activities and mitigates the HMs’ mobility (Ullah et al., 2020; Rizwan et al., 2021). Biochar has excellent adsorption capacity, and it adsorbs a significant amount of HMs, thereby reducing the toxic effects of HMs (Jia et al., 2017).

The efficiency of BC to remediate polluted soils can be improved by its combined use with different amendments—for instance, the use of BC in combination with compost provided better results in terms of improved soil fertility, water holding capacity, nutrient uptake, and HM adsorption (Agegnehu et al., 2015; Coelho et al., 2018). Thus, we hypothesized that BCC could be an effective amendment to improve growth, physiology, antioxidant activities, and soil properties by mitigating the harmful impacts of Pb. The study was performed with the following objectives: (i) to determine the impacts of BC, compost, and BCC on the productivity, oil yield, and physiological and biochemical functioning of *Brassica* and (2) to determine the impacts of compost, BC, and BCC on soil properties and their ability to reduce the HR of *Brassica* grown in Pb-polluted soil.

## Materials and methods

The soil was collected from the ricefield of Hunan University of Humanities, Science, and Technology. The soil was collected from the 0–20-cm soil layer, and analysis of the collected soil was done to determine the different soil properties. The soil had a silty loam texture with pH of 5.41, cation exchange capacity of 7.41 centimoles (cmol) kg^-1^, organic carbon of 11.62 g kg^-1^, and available phosphorus and potassium of 26.33 and 108.13 mg kg^-1^, respectively.

### Preparation of biochar, compost, and biochar co-compost

To prepare BC, rice straws were collected, and pyrolysis was done for 8 h at 600°C to prepare BC. Thereafter, BC was sieved through a 2-mm sieve and used to determine the different properties. Biochar had an alkaline nature (pH: 9.9), with 640 g kg^-1^ carbon content, 10.97 centimoles (cmol) kg^-1^ CEC, and 4.52 g kg^-1^ N content. The compost used in the study was prepared by using animal manure with a composting unit. The composting process was carried out for 40 days under the following conditions: 50% moisture, 65°C temperature, and 50 rpm speed. Moreover, during the last week of composting, BC (30%: WW) was mixed with composted material to prepare BCC. The compost had 6.98 pH, 251 g kg^-1^ carbon content, 7.91 cmol kg^-1^ CEC, and 2.98 N g kg^-1^ content. Moreover, BCC had 8.2 pH, 498 g kg^-1^ carbon content, 10.33 cmol kg^-1^ CEC, and 4.12 g kg^-1^ N contents.

### Experimental treatments

The study comprised of the following treatments—control and Pb stress 100 mg kg^-1^—and organic amendments—control, biochar, compost, and biochar co-compost (BCC). The biochar, compost, and BCC were used at the rate of 2% (W/W). The pots (38 cm × 34 cm) were filled with 10 kg soil, and Pb salt was thoroughly mixed with the soil. The soil was aged for 4 weeks before using it for the experiment. After 4 weeks, biochar, compost, and BCC were applied at the rate of 2% (W/W) and thoroughly mixed with the soil. Every amendment was applied at a rate of 200 g in 10 kg soil to achieve the 2% rate of application. A total of 10 seeds of *Brassica napus* were sown in each pot, and after germination five plants were tinned. The study was performed in an open greenhouse equipped with a rain-shed to prevent interference from rainwater. The pots were regularly visited to fulfill irrigation requirements, while other agronomic practices were kept constant to get a good stand establishment. The plants were harvested at the physiologically mature stage to determine different growth and yield traits. Moreover, samples were collected after 30 days of sowing to determine different physiological and biochemical traits.

### Measurement of growth traits

Five plants from each pot were carefully uprooted from each pot. The roots were separated from shoots to determine their fresh weights. Then, both roots and shoots were oven-dried (65°C) until constant weight to assess their dry weights.

### Determination of chlorophyll content and leaf water status

Fresh leaf samples (0.5 g) were ground in 80% acetone, and the extract was collected. Thereafter, absorbance was measured (663, 645, and 480 nm) to determine the concentration of chlorophyll a and b and carotenoids (Arnon, 1949). To determine relative water contents (RWC), fresh top leaf samples were collected, and their fresh weight (FW) was taken. Then, they were dipped in water for 24 h, and their turgid weight (TW) was measured. The samples were dried until constant weight (DW), and finally, RWC was assessed by using the following method: RWC = (FW − DW)/(TW − DW) × 100 (Chattha et al., 2022). In case of electrolyte leakage: we took fresh leaves (0.5 g) and soaked them in water for 24 h, and EC1 was measured. The same leaves were placed in a water bath for a period of 2 h, and a second EC2 was taken. The final EL was assessed using the following method: EL% = (EC1/EC2) × 100.

### Determination of osmolytes and oxidative stress markers

To determine the concentration of total soluble proteins (TSP), 0.5 g fresh leaf samples was collected and homogenized in 5 mL of potassium phosphate buffer (PPB; pH: 7.8), and the extract was collected. The obtained extract was centrifuged for 15 min at 14,000 rpm. Thereafter, 2 mL of Bradford was added to the extract and placed at room conditions for 20 min, and the absorbance (595 nm) was taken to determine the concentration of TSP (Bradford, 1976). To determine the concentration of free amino acids, fresh leaf samples (0.5 g) were taken and ground with 5 mL PPB solution to obtain the extract. The extract was mixed with 1 mL of pyridine and ninhydrin, boiled for 30 min in a water bath (90°C), and allowed to cool, and the absorbance (570 nm) was noted (Hamilton and Van-Slyke, 1943). Plant sample (0.5 g) was collected and extracted using 10 mL sulpho-salicylic acid (3%). The extract was centrifuged for 10 min at 10,000 rpm. Then, the supernatant was incubated for 30 min at 90°C in a water bath after mixing with 2 mL of glacial acetic acid and ninhydrin. The mixture was cooled, toluene was added to the extract, and proline contents were determined by measuring the absorbance at 520 nm (Bates et al., 1973). To assess both stress markers (H_2_O_2_ and MDA), fresh leaf samples (0.5 g) were ground in tri-chloroacetic acid (5 mL), and the extract was obtained. To measure H_2_O_2_, 1 mL extract was mixed with 1 mL of PBB, and absorbance was taken at 390 nm (Velikova et al., 2000). For the MDA concentration, the extract was centrifuged for 15 min at 12,000 rpm and thereafter mixed with 5 mL thiobarbituric acid and boiled for 30 min. Its absorbance was taken to determine MDA concentration (Rao and Sresty, 2000).

### Determination of antioxidant activities

The standard procedure of Nakano and Asada (1987) was used to determine the concentration of APX. For this, 0.5 g fresh leaf was homogenized using 5 mL of PPB and centrifuged at 10,000 rpm for 4 min, and absorbance (290 nm) was taken. The fresh samples of leaf (0.5) were ground with 5 mL of PPB and centrifuged (10,000 rpm) for 10 min, and absorbance was recorded at 240 and 470 nm to determine CAT and POD activities using the methods of Aebi (1984) and Zhang (1992). In the case of SOD activity, a reaction mixture containing 100 µL H_2_O_2_, 25 mL buffer, 100 µL Triton, 50 µL sample, and 50 µL riboflavin was prepared, and absorbance was measured at 560 nm (Zhang, 1992).

### Determination of plant tissue nutrient concentration and soil properties

After harvesting, *Brassica* plant samples were ground and digested with two acids (HCl and HNO_3_) in 1:2 ratio. Then, the samples were filleted and diluted by using water, and the concentration of N was measured by using the Kjeldahl procedure, while the concentration of P was determined using a spectrophotometer. The concentration of Ca, Mg, and K was measured by using a flame photometer. Soil N was determined by using the Kjeldahl method, while P and K were measured by sodium bicarbonate and ammonium acetate extraction methods, respectively. We took 20 g of soil and fumigated it for 24 h with chloroform. Thereafter, both fumigated as well as non-fumigated soils were extracted with 0.5 M K_2_SO_4_ solution, and microbial biomass carbon (MBC) concentration was measured with a carbon analyzer.

### Evaluation of phyto-immobilization efficiency

The bioaccumulation concentration (BAC) and bio-accumulation factor (BAF) were assessed by using the proposed methods of Amin et al. (1954) and Zhuang et al. (2007). BAC was measured by using the following equation—BAC = Pb (shoot)/Pb (soil)—while BCF was determined with this formula: BCF = M (harvested tissue)/M (water). Here M is the concentration of Pb in plant parts (root, shoot, and grains), while M (soil) is the total amount of metal (Pb) applied according to treatments.

### Intake of metal and assessment of health risks

The daily intake of metal (DIM) was determined by using the methods of Latif et al. (2018) with the following equation: DIM = M × I × K/W. Here in this equation, M is Pb concentration in plant parts (mg kg^-1^), K indicates conversion factor, while I and W are daily intake of vegetable and average body weight, respectively. The average daily vegetable intake per person was 0.345 kg, while the average body weight was 60 kg (Latif et al., 2018). Moreover, the HR index was calculated using the standard methods of Jan et al. (2010): HRI = DIM/Rfd. In the aforementioned equation, Rfd indicates reference oral dose which is 0.02 mg kg^-1^, while DIM is the daily intake of metal (United States Environmental Protection Agency, Integrated Risk Information System: US-EPA IRIS).

### Determination of yield traits and seed oil contents

The brassica plants were harvested at the maturity stage to determine different traits like branches per plant, pod length, and seeds per pod. The pods from each plant were separated and threshed to determine the seed yield per pot. Moreover, a sub-sample of seeds was taken, and oil concentration was determined by the Soxhlet apparatus (AOAC, 1990).

### Data analysis

The collected data were analyzed with a two-way analysis of variance using a computer-based software (Statistix 8.1). The least significant difference (LSD) at 5% probability was used to determine the significance among means. The figures were made with sigma-plot 10, while PCA was performed with R-studio.

## Results

### Growth traits

Lead stress caused a marked reduction in the growth traits of *Brassica*. Nonetheless, BC and BCC reversed the toxicity of Pb and improved *Brassica* growth. The application of BCC resulted in a significant increase in plant height (24.1%), RL (37.82%), RFW (41.81%), and RDW (56.34%) under no Pb stress. Opposite to this, BCC improved the plant height, RL, RFW, and RDW by 15.23%, 48.52%, 43.72%, and 56.30%, respectively, under Pb stress (**Table 1**).

**Table 1:** Effect of biochar, compost and biochar co-compost on the growth traits of brassica grown under lead stress.

Treatments		PH (cm)	RL (cm)	RFW (g)	RDW (g)	BPP
**Control**	**Control**	128c ± 1.24	34.37c ± 1.71	27.50c ± 1.43	14.50cd ± 0.41	5.33
	**BC**	145b ± 3.29	43.72ab ± 0.91	38.33a ± 1.72	19.34b ± 0.98	6.67
	**Compost**	145b ± 2.49	40.35b ± 1.68	33.68b ± 1.23	16.36c ± 1.10	5.33
	**BCC**	159a ± 2.99	47.37a ± 1.88	39.00a ± 2.16	22.67a ± 0.95	7.33
**Pb stress**	**Control**	105e ± 3.65	20.40e ± 2.24	16.33e ± 2.89	8.17f ± 0.84	5.33
	**BC**	113de ± 2.94	27.00d ± 1.17	21.69d ± 3.45	10.33ef ± 0.93	5.67
	**Compost**	108e ± 4.16	22.01e ± 0.88	19.33de ± 1.24	9.00f ± 0.99	5.33
	**BCC**	121cd ± 3.85	30.03d ± 1.43	23.47cd ± 0.75	12.77de ± 0.80	5.67

The data is mean (*n* = 3) ± SE, and different letters show the significance between means. The effect of different treatments on BPP was non-significant; therefore, no lettering and SE were applied.

Pb, lead; BC, biochar; BCC, biochar co-compost; PH, plant height; RL, root length; RFW, root fresh weight; RDW, root dry weight; BPP, branches per plant.

### Photosynthetic pigments and leaf water status

**Figure 1:**
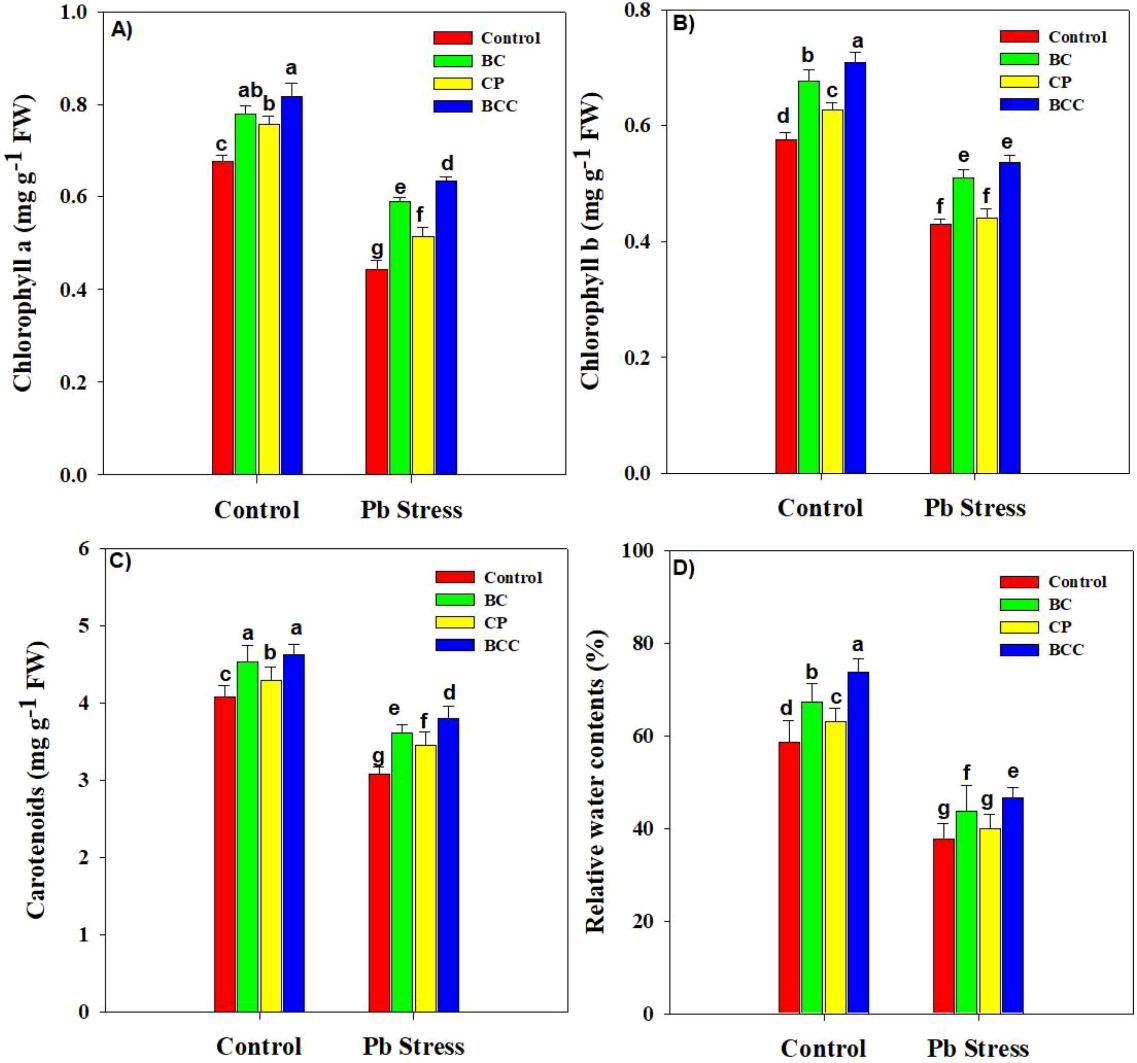
Effect of biochar, compost, and biochar co-compost on chlorophyll a **(A)**, chlorophyll b **(B)**, carotenoid **(C)**, and relative water contents **(D)** of *Brassica* grown under Pb stress. The bars indicate means with standard errors (*n* = 3), and bars with the same letter(s) show the non-significant differences with each other, *p* < 0.05.

Lead stress significantly reduced the RWC and chlorophyll contents. BCC increased the chlorophyll a, chlorophyll b, carotenoid, and RWC by 20.58%, 22.41%, 13.20%, and 25.56% under normal conditions. Moreover, the same treatment increased chlorophyll a, chlorophyll b, carotenoid, and RWC by 43.18%, 25.58%, 23.30%, and 23.89% under Pb stress (**Figure 1**).

### Oxidative stress markers, antioxidant activities, and osmolyte accumulation

**Figure 2:**
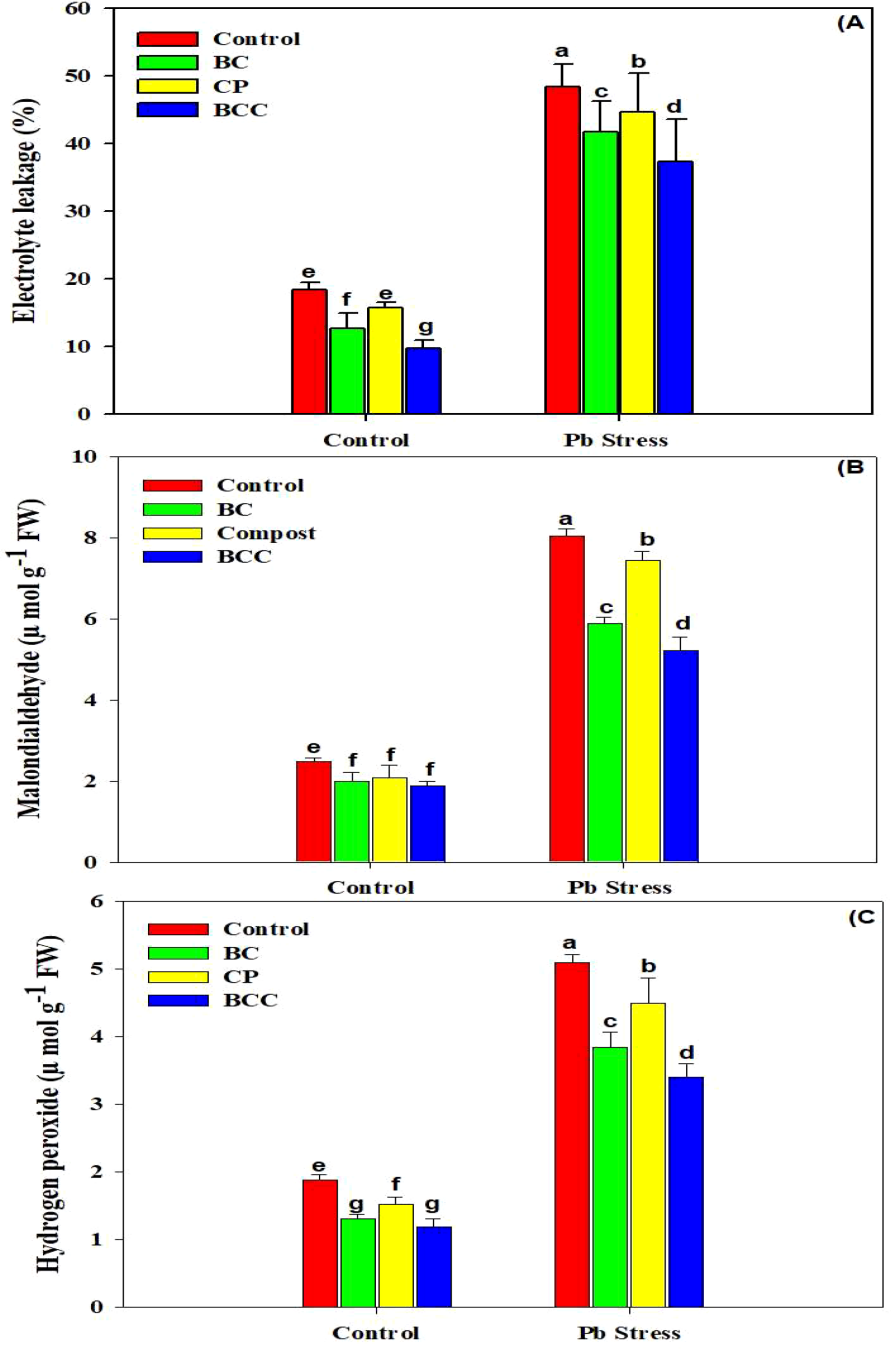
Effect of biochar, compost, and biochar co-compost on electrolyte leakage **(A)**, malondialdehyde **(B)**, and hydrogen peroxide **(C)** of *Brassica* grown under Pb stress. The bars indicate means with standard errors (*n* = 3), and bars with the same letter(s) show the non-significant differences with each other, *p* < 0.05.

Three oxidative stress markers (EL, MDA, and H_2_O_2_) showed a significant increase under Pb stress (**Figure 2**). Biochar, compost, and especially BCC mitigated the accumulation of the aforesaid oxidative stress markers, and the overall trend of different treatments in decreasing the oxidative stress was observed as follows: BCC > BC > compost > control. The four antioxidant enzymes (APX, CAT, POD, and SOD) showed an increase in their activity under Pb stress. The minimum activity of these antioxidants was observed in control soil. The activity of the antioxidants was increased under Pb stress, indicating that *Brassica* plants activated their antioxidant activities to counter the toxic effects of Pb stress.

Biochar treatments increased the antioxidant activities, and maximum APX, CAT, POD, and SOD activity was recorded in BCC followed by BC and compost under Pb stress conditions (**Table 2**). Pb stress determined variable effects on the osmolyte accumulation—for instance, TSP (59.63%) and FAA (73.60%) were decreased under Pb stress conditions, while proline concentration (21.21%) was increased under Pb stress (**Table 2**). Different amendments increase osmolyte synthesis, though BCC remained the top performer in increasing the osmolyte concentration under Pb stress, followed by BC and compost (**Table 2**).

**Table 2:** Effect of biochar, compost and biochar co-compost on the osmolyte accumulation and antioxidant activities of brassica grown under lead stress.

Treatments		TSP (mg/g FW)	FAA (mg/g FW)	Proline (mg/g FW)	APX (U/mg protein)	CAT (U/mg protein)	POD (μ/mg protein)	SOD (U/mg protein)
**Control**	**Control**	15.22c ± 0.46	11.73d ± 0.44	0.61e ± 0.022	18.83f ± 0.94	4.02f ± 0.12	1.88f ± 0.056	1.88g ± 0.076
	**BC**	17.01b ± 1.12	16.40b ± 0.67	0.69d ± 0.033	26.77de ± 1.24	5.60d ± 0.21	2.79de ± 0.098	3.19e ± 0.089
	**Compost**	16.29b ± 0.89	15.00c ± 0.76	0.65de ± 0.044	24.27e ± 1.06	5.00e ± 0.19	2.44e ± 0.12	2.76f ± 0.099
	**BCC**	18.12a ± 0.78	17.91a ± 0.55	0.70cd ± 0.012	30.21d ± 0.98	6.22c ± 0.18	3.12d ± 0.061	3.54d ± 0.076
**Pb stress**	**Control**	9.59f ± 0.65	7.82f ± 0.49	0.71cd ± 0.021	34.30c ± 1.54	6.86b ± 0.42	4.05c ± 0.087	4.05e ± 0.112
	**BC**	10.48e ± 0.91	9.03ef ± 0.67	0.82b ± 0.019	43.57b ± 1.76	8.86a ± 0.10	4.48b ± 0.220	4.88b ± 0.119
	**Compost**	9.84ef ± 0.74	8.44f ± 0.33	0.75c ± 0.045	37.30c ± 1.28	7.25b ± 0.22	4.15bc ± 0.141	4.27c ± 0.220
	**BCC**	11.81d ± 0.52	9.89e ± 0.41	0.93a ± 0.052	48.33a ± 0.99	9.11a ± 0.19	5.06a ± 0.141	5.40a ± 0.118

The data is mean (*n* = 3) ± SE, and different letters show the significance between means.

Pb, lead; BC, biochar; BCC, biochar co-compost; TSP, total soluble proteins; FAA, free amino acids; CAT, catalase; POD, peroxidase; SOD, superoxide dismutase.

### Tissue nutrient concentration

Lead stress determined a sharp decrease in the concentration of all the nutrients. Conversely, addition of BCC significantly increased the N, Ca, Mg, and K concentration in *Brassica* tissues. The overall trend of different BC treatments in increasing the concentration of N, Ca, Mg, and K was observed as follows: BCC > BC > compost > control (**Table 3**).

**Table 3:** Effect of biochar, compost and biochar co-compost on the tissue nutrient concentration of brassica grown under lead stress.

Treatments		Nitrogen (mg g^-1^ DW)	Calcium (mg g^-1^ DW)	Magnesium (mg g^-1^ DW)	Potassium (mg g^-1^ DW)
**Control**	**Control**	13.27d ± 0.88	46.35d ± 1.71	36.17c ± 1.88	17.58c ± 0.98
	**BC**	19.62b ± 1.12	54.17b ± 1.75	44.00b ± 1.45	24.00b ± 1.45
	**Compost**	17.16c ± 1.33	49.70c ± 1.45	39.75c ± 1.20	22.37b ± 1.98
	**BCC**	21.21a ± 2.19	57.94a ± 1.03	48.28a ± 0.84	28.05a ± 2.44
**Pb stress**	**Control**	8.65g ± 0.87	29.03g ± 1.41	22.20f ± 1.45	13.96d ± 1.20
	**BC**	10.43ef ± 1.31	34.37f ± 1.23	27.32e ± 2.22	14.89d ± 1.77
	**Compost**	9.08fg ± 0.45	31.63fg ± 1.98	25.23ef ± 2.45	14.14d ± 2.66
	**BCC**	11.85de ± 1.56	37.53e ± 2.10	31.60d ± 1.98	15.58cd ± 2.34

The data is mean (*n* = 3) ± SE, and different letters show the significance between means.

Pb, lead; BC, biochar; BCC, biochar co-compost.

### Yield and yield traits and oil contents

Brassica yield and yield traits were significantly reduced under Pb stress, while BC, compost, and BCC significantly increased the yield traits and oil contents of *Brassica*. The maximum PPP (214.68), PL (5.85 cm), SPP (22.04), and SYPP (13.83 g) were recorded with BCC application, followed by BC and compost in control soil. Under Pb stress conditions, maximum PPP (137.67), PL (4.47 cm), SPP (12.10), and SYPP (8.65 g) were observed with BCC application (**Table 4**). The oil contents of *Brassica* were significantly decreased under Pb stress, though the application of BC treatments mitigated the adverse impacts of Pb and improved the oil concentration. Pb stress caused a reduction of 21.36% in oil contents compared to control conditions. The application of BCC increased the oil contents by 11.06%, while BC and compost increased the oil contents by 10.90% and 5.14%, respectively (**Table 4**).

**Table 4:** Effect of biochar, compost, and biochar co-compost on yield traits and oil contents of brassica grown under lead stress.

Treatments		Pods per plant	Pod length (cm)	Seeds per pod	Seed yield per pot (g)	Oil contents (%)
**Control**	**Control**	171.67d ± 5.31	4.86c ± 0.22	15.63c ± 0.82	10.16c ± 0.45	38.17c ± 1.78
	**BC**	202.00b ± 6.12	5.12b ± 0.14	20.17ab ± 0.78	12.03b ± 0.56	40.53ab ± 2.33
	**Compost**	189.83c ± 4.56	5.05bc ± 0.19	17.83bc ± 0.23	11.78b ± 0.94	39.08bc ± 1.99
	**BCC**	214.68a ± 7.55	5.84a ± 0.21	22.04a ± 0.57	13.83a ± 0.44	41.32a ± 2.24
**Pb stress**	**Control**	111.33f ± 2.63	4.14e ± 0.39	8.73e ± 0.99	6.16e ± 0.28	30.72e ± 2.69
	**BC**	131.73e ± 4.09	4.28de ± 0.12	11.30de ± 0.49	7.88d ± 0.11	34.07d ± 1.76
	**Compost**	116.70f ± 1.75	4.22de ± 0.19	9.30e ± 1.12	6.61e ± 0.47	32.20e ± 2.33
	**BCC**	137.67e ± 2.99	4.47d ± 0.26	12.10d ± 1.41	8.65d ± 0.59	34.12d ± 3.12

The data is mean (*n* = 3) ± SE, and different letters show the significance between means.

Pb, lead; BC, biochar; BCC, biochar co-compost.

### Tissue Pb concentration

**Figure 3:**
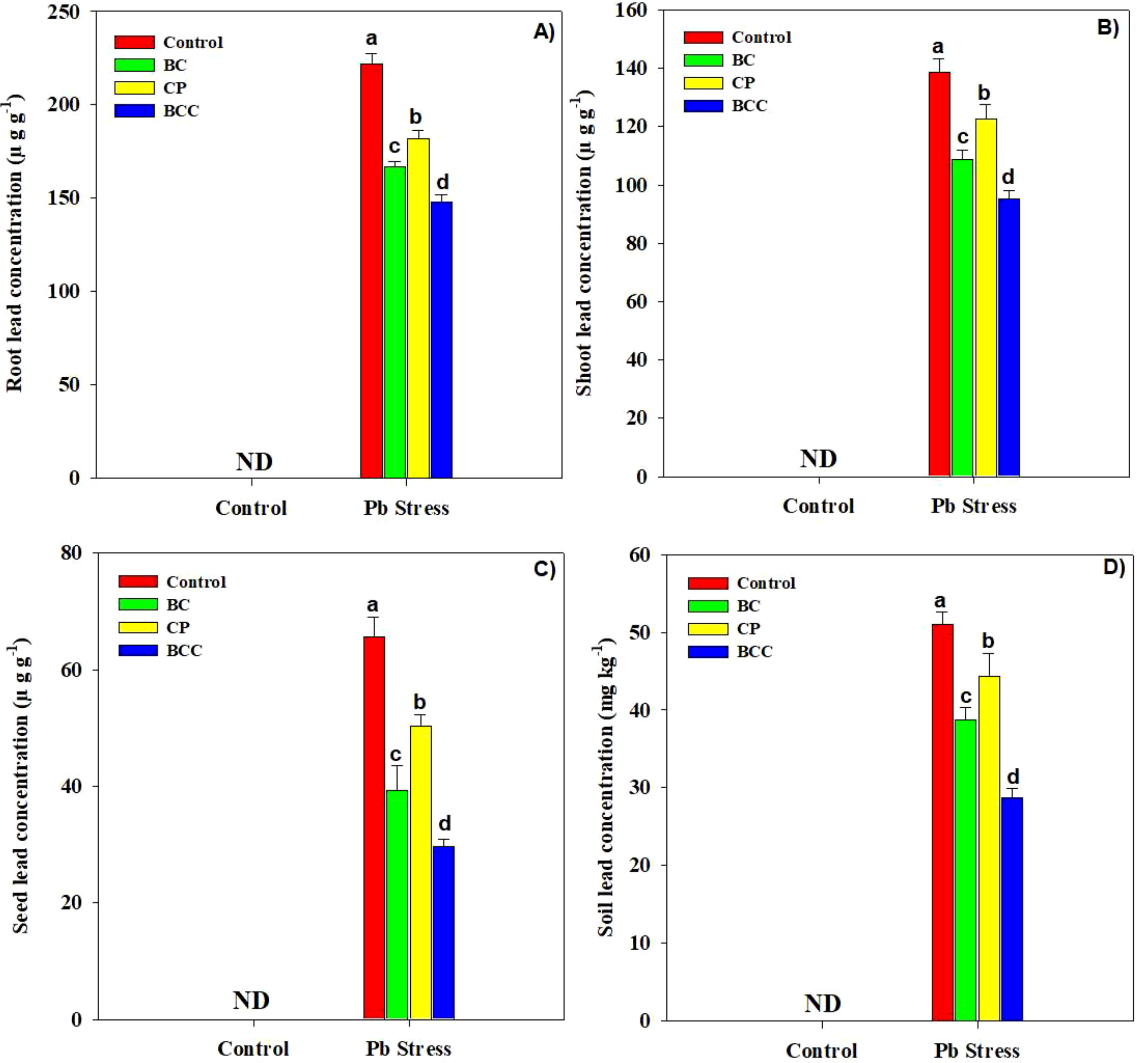
Effect of biochar, compost, and biochar co-compost on Brassica napus root **(A)**, shoot **(B)**, seed **(C)**, and soil Pb concentration **(D)**. ND: not detected. The bars indicate means with standard errors (*n* = 3), and bars with the same letter(s) show the non-significant differences with each other, *p* < 0.05.

The accumulation of Pb was significantly increased in root, shoot, and seed samples of *Brassica* in Pb-polluted soil. Conversely, BCC, BC, and compost decreased the Pb concentration in plant organs. The application of BC, compost, and BCC reduced the Pb concentration by 33.08%, 45.46%, and 121.33% (**Figure 3**).

### Soil properties

The results indicated that Pb and the application of different BC treatments significantly affected the soil properties. The application of BCC, BC, and compost significantly increased the soil pH, total N, and available P and K. Maximum soil pH (5.93), total N (1.33 g kg^-1^), available P (25.34 mg kg^-1^), and available K (97.77 mg kg^-1^) under control were noted with the application of BCC, followed by BC and compost. Furthermore, maximum soil pH (5.61), total N (1.07 g kg^-1^), available P (17.97 mg kg^-1^), and available K (79.22 mg kg^-1^) in Pb-containing soil was observed with BCC application. Moreover, Pb stress showed a reduction of 18.39% and 18.84% in SOC and MBC. Biochar and BCC treatments significantly increased both SOC and MBC (**Table 5**).

**Table 5:** Effect of biochar, compost, and biochar co-compost on soil properties.

Treatments		pH	Total N (g kg^-1^)	Available P (mg kg^-1^)	Available K (mg kg^-1^)	SOC (mg kg^-1^)	MBC (mg kg^-1^)
**Control**	**Control**	5.68c ± 0.091	1.13cd ± 0.069	19.27cd ± 1.15	81.80cd ± 3.77	15.83d ± 0.99	315.00f ± 7.98
	**BC**	5.84ab ± 0.11	1.27ab ± 0.045	23.33b ± 0.99	92.80ab ± 4.45	19.98b ± 1.12	441.33ab ± 11.22
	**Compost**	5.74bc ± 0.17	1.19bc ± 0.056	20.67b ± 2.33	88.37bc ± 4.98	18.04c ± 1.45	432.67b ± 9.31
	**BCC**	5.93a ± 0.082	1.33a ± 0.024	25.34a ± 1.78	97.77a ± 5.02	21.86a ± 0.89	454.00a ± 17.66
**Pb stress**	**Control**	5.38e ± 0.045	0.81f ± 0.046	14.82g ± 2.42	5.37f ± 1.34	15.11f ± 0.45	300.00f ± 15.44
	**BC**	5.49de ± 0.13	0.97e ± 0.033	16.80ef ± 3.33	75.73de ± 5.33	16.18ef ± 0.87	361.67e ± 18.44
	**Compost**	5.41e ± 0.15	0.86f ± 0.031	15.73fg ± 1.65	69.28ef ± 2.99	15.72ef ± 0.92	339.33f ± 14.55
	**BCC**	5.61cd ± 0.018	1.07de ± 0.045	17.97de ± 1.41	79.22d ± 3.87	16.92de ± 1.09	380.33c ± 16.78

The data is mean (*n* = 3) ± SE, and different letters show the significance between means.

Pb, lead; BC, biochar; BCC, biochar co-compost; N, nitrogen; P, phosphorus; K, potassium; SOC, soil organic carbon; MBC, microbial biomass carbon.

### Phyto-stabilization efficiency and health risk assessment

**Figure 4:**
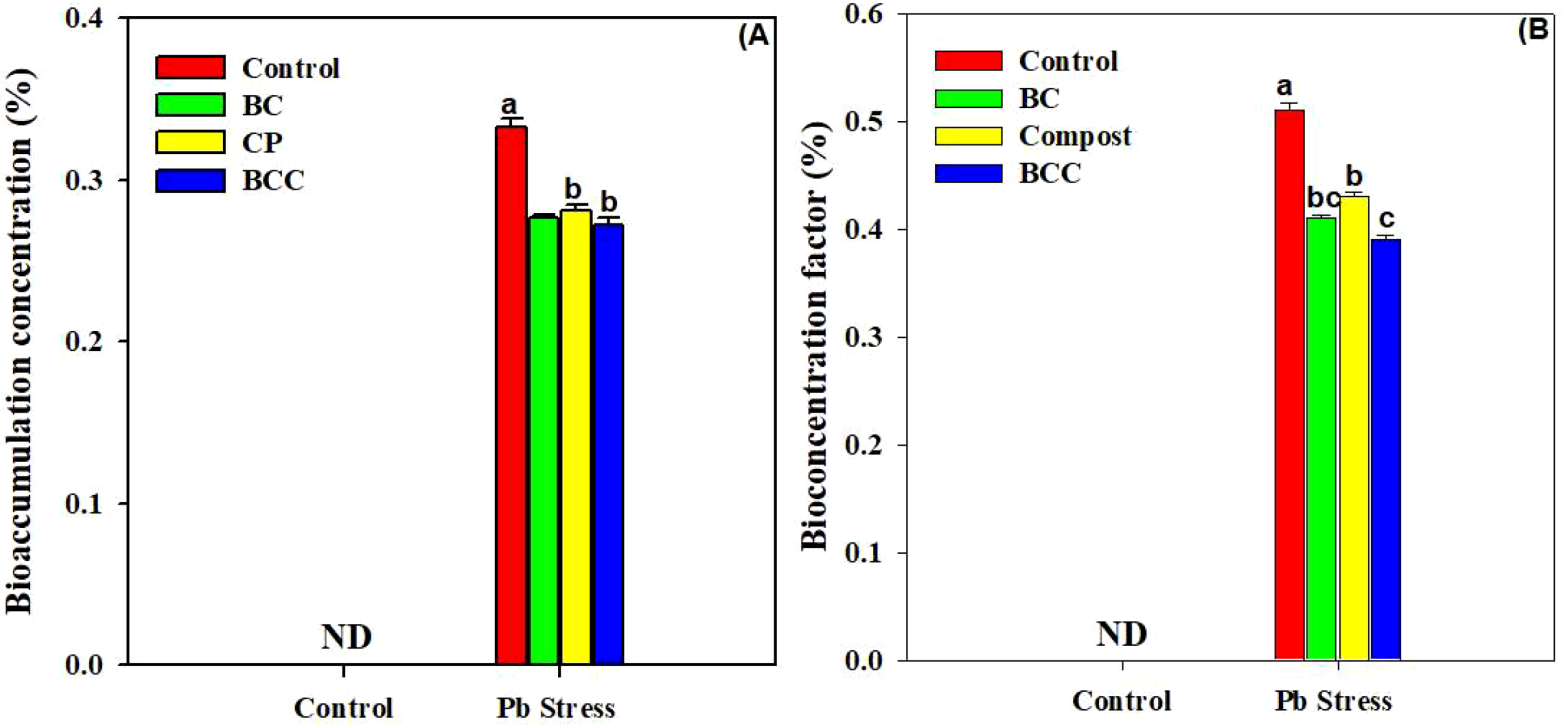
Effect of biochar, compost, and biochar co-compost on bioaccumulation concentration **(A)** and bio-concentration factor **(B)** of Brassica grown under Pb stress. ND: not detected. The bars indicate means with standard errors (*n* = 3), and bars with the same letter(s) show non-significant differences with each other, *p* < 0.05.

**Figure 5:**
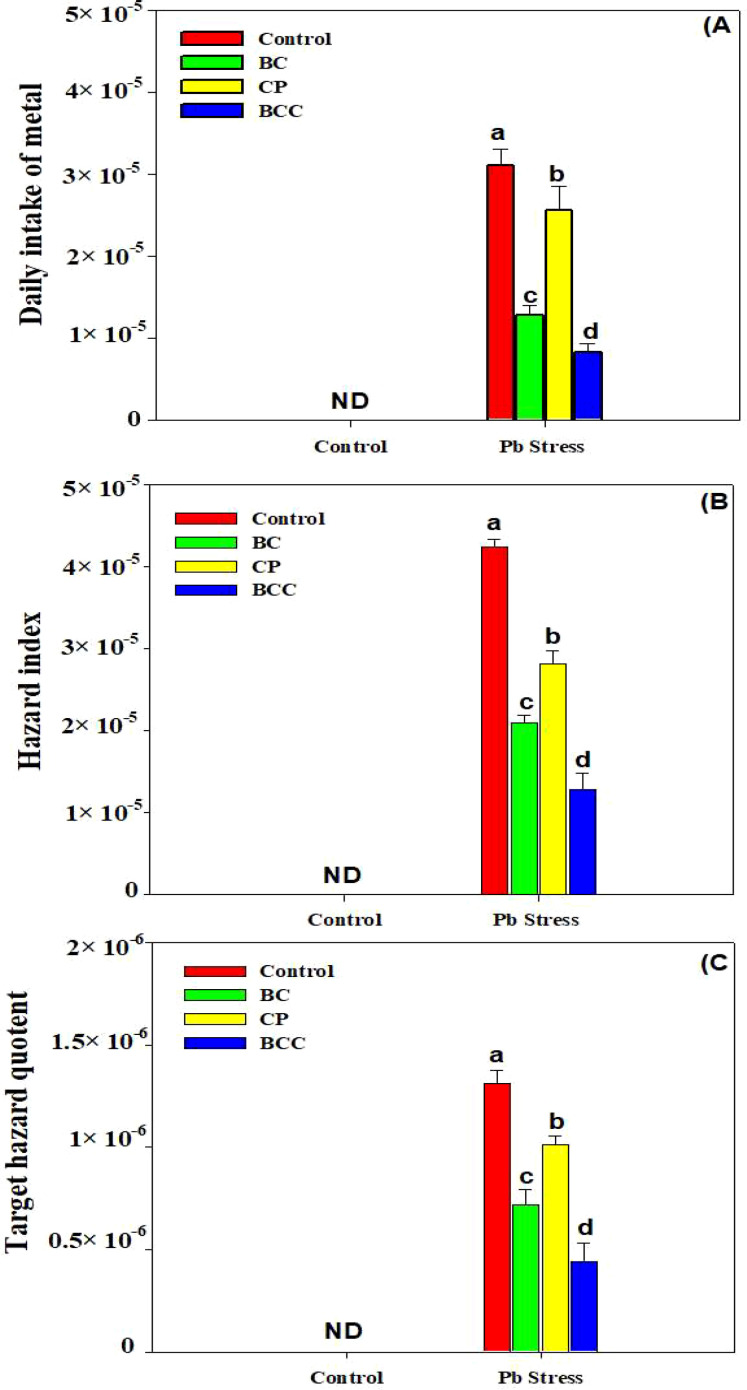
Effect of biochar, compost, and biochar co-compost on daily metal intake **(A)**, hazard index **(B)**, and target hazard quotient **(C)**. ND: not detected. The bars indicate means with standard errors (*n* = 3), and bars with the same letter(s) show the non-significant differences with each other, *p* < 0.05.

The application of BC, compost, and particularly BCC showed appreciable potential to phyto-stabilize Pb compared to the control. Minimum BAC and BAF were observed with the application of BCC, followed by BC and compost (**Figure 4**). A significant reduction in HR linked with edible parts of *Brassica* was also observed by using BC and BCC. The soil treated with BCC showed minimum DIM, HI, and THQ compared to the other treatments (**Figure 5**).

### Principle component analysis

**Figure 6:**
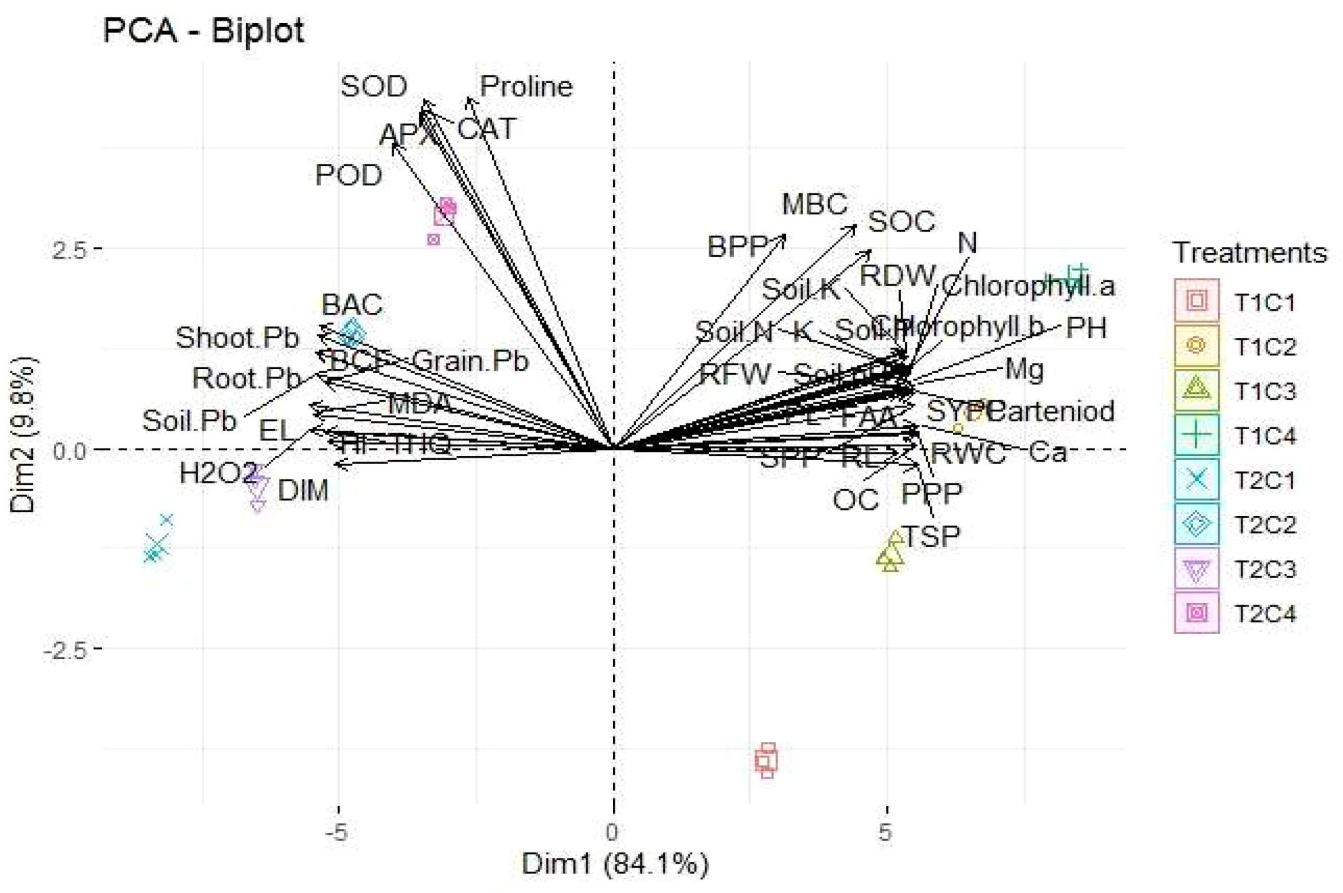
Loading plot on the right and scores on the left side of PCA, indicating the impact of different treatments on the studied parameters.

According to the PCA results, the first PCA (PC1) has a major share of 81.1%, while the second PCA (PC2) has a minor share of 9.8%. The results indicated a positive link between CAT, POD, SOD, proline, BAC, shoot-Pb, EL, MDA, SOC, MBC, BPP, soil nutrients, FAA, TSO, SOC, and RWC and a negative association between DIM and H_2_O_2_ (**Figure 6**).

## Discussion

**Figure 7:**
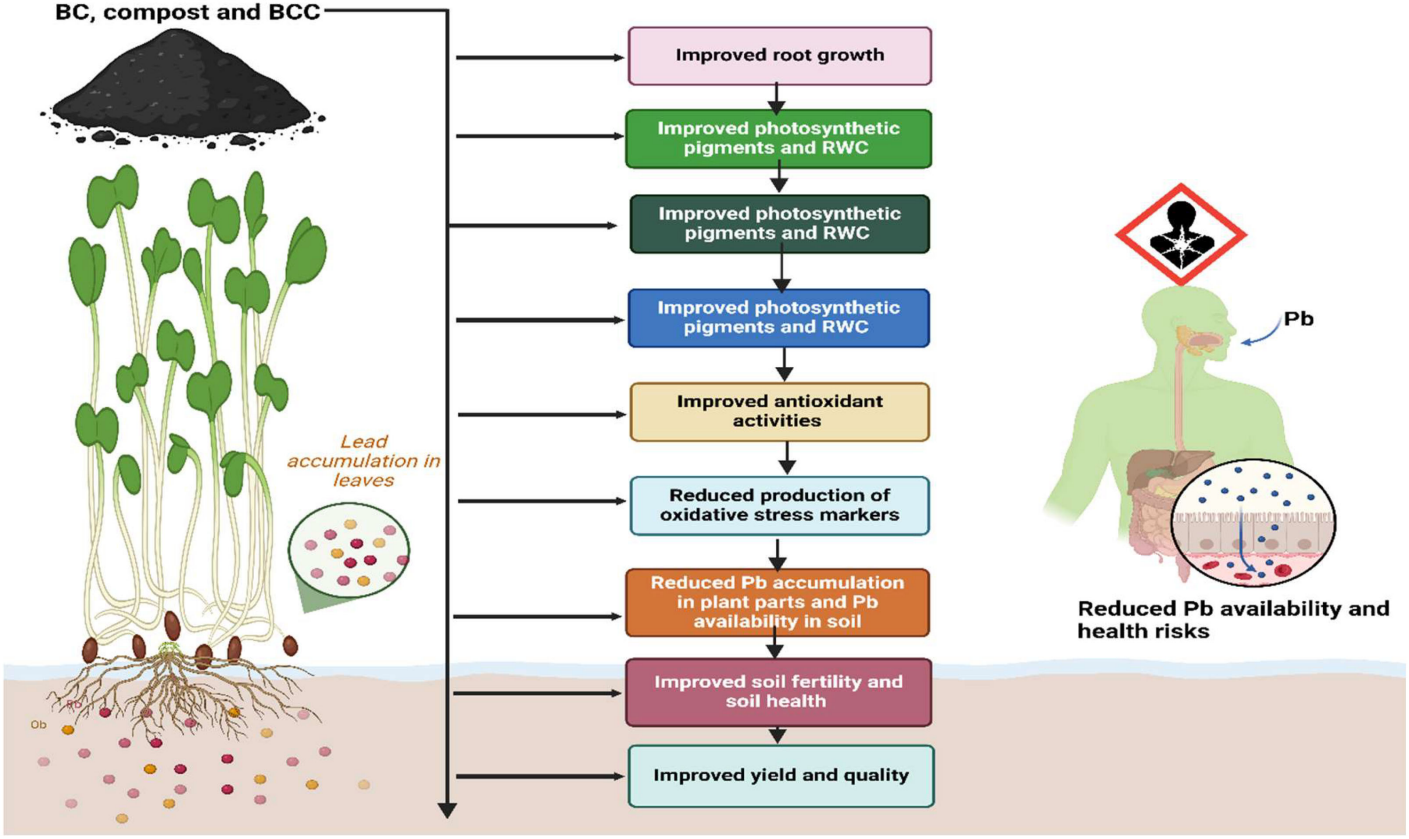
Schematic diagram indicating the effect of different amendments on brassica under Pb stress. The application of BC, compost, and BCC improves root growth, photosynthetic pigments, leaf water contents, antioxidant activities, and soil health and reduces Pb availability, thereby leading to safer and better production.

Lead toxicity caused a marked reduction in the growth traits of *Brassica* plants (**Table 1**) which was linked with an increase in EL, MDA, and H_2_O_2_; **Figure 7**) and a reduction in the synthesis of chlorophyll contents and leaf water status (Muradoglu et al., 2015; El-Shora et al., 2021; Osman and Fadhlallah, 2023). The application of BCC significantly improved the growth of *Brassica* plants by increasing SOC, nutrient retention (**Table 5**), leaf water status, and chlorophyll synthesis and reducing Pb uptake and accumulation (Ali et al., 2018; Novak et al., 2019; Abbas et al., 2020; Cheng et al., 2020). The increase in SOC increases nutrient availability, their uptake, and microbial and enzyme activity, which lead to improved plant growth (Gerke, 2022). In the present study, all of the amendments, particularly BCC, significantly increased both SOC and MBC and plant growth, and it was further proved by a positive relationship between SOC, MBC, and growth and yield (**Figure 6**). Chlorophyll is an important photosynthetic pigment that absorbs light energy in photosynthesis (Hu et al., 2012). In this experiment, Pb stress significantly reduced chlorophyll synthesis in *Brassica*. This was likely due to increased MDA and H_2_O_2_ that damaged the photosynthetic apparatus by increasing oxidative stress and increasing the activity of chlorophyll-degrading enzymes (El-Shora et al., 2021; Qin et al., 2023). The lead-induced increase in H_2_O_2_ might disrupt the enzymes’ activity involved in chlorophyll synthesis and accelerated chlorophyll degradation, thereby leading to a significant reduction in chlorophyll synthesis (Dalyan et al., 2020). The addition of BCC significantly increased the chlorophyll and carotenoid contents of *Brassica* plants. Biochar alleviated the inhibitory effects of Pb by decreasing its concentration in soil, which reduced the oxidative damages to photosynthetic apparatus, resulting in better chlorophyll and carotenoid synthesis under HM stress (Haider et al., 2022).

Pb stress significantly increased the MDA and H_2_O_2_ concentration which caused the membrane damage as indicated by a higher electrolyte leakage concentration (**Figure 2**). This is consistent with earlier findings of different authors who also found that HMs increased the MDA concentration owing to ROS-induced oxidative burst (Jawad et al., 2020; Cheng et al., 2021). The antioxidants (APX, CAT, POD, and SOD) were profoundly enhanced under Pb stress (**Table 2**). SOD is an important antioxidant enzyme responsible in converting superoxide radicals into H_2_O_2_, and it plays an important role to protect the cell from the damaging effects of ROS. In this study, plants grown under Pb stress showed an increase in SOD activity, which was likely due to the *de novo* synthesis of enzyme proteins because of an increase in synthesis of superoxide radicals (Naqqash et al., 2022). CAT is also an important antioxidant that eliminates H_2_O_2_ by converting it into H_2_O (Noctor and Foyer, 1998). The increased CAT activity under Pb stress conditions can be attributed to increased H_2_O_2_ production under stress conditions (Shabaan et al., 2021). BCC and BC significantly increased the activities of all the four antioxidants, which helped the brassica plants alleviate the toxic impacts of Pb. The maximum concentration of Pb was found in roots than in shoots, and a little concentration of Pb was reported in the seeds of *Brassica*. The application of BCC significantly reduced the concentration of Pb in all plant tissues (**Figure 3**). Biochar has a higher surface area that facilitates sorption, ion exchange, complexation, precipitation, and coprecipitation of HMs, thereby reducing their availability and subsequent accumulation in plant tissues (Cheng et al., 2020; Mehmood et al., 2021; Murtaza et al., 2021). BCC significantly decreased the availability of Pb in soil (**Table 5**), which also resulted in less Pb concentration in plant tissues (**Figure 3**). This significant reduction in plant parts by BCC could also be related to improved immobilization of Pb and the dilution effect as a result of increased biomass production (**Table 1**). Lead toxicity also reduced the yield and oil concentration of *Brassica* plants, which was linked with decreased chlorophyll contents, RWC, total soluble protein, and free amino acid accumulation and increased MDA, EL, and H_2_O_2_. Biochar co-compost significantly increased the soil pH due to the presence of alkali ions in BCC that absorbed H^+^ and exchangeable Al^3+^ from the soil, resulting in an increase in soil pH (Xu et al., 2020; Ma et al., 2021). The presence of alkaline ions, oxide, and hydroxide in BC increases the soil pH, which facilitates the precipitation and fixation of HMs, thus reducing their concentration (**Table 5**) and biological effectiveness (Bashir et al., 2020). The aforementioned functional groups on the BC surface might also chelate the metals and play an important role in the complexation of HMs on the surface as well as inner pores of BC. The availability of Pb in soil (**Table 5**) was significantly decreased after BCC and BC application, which was another possible reason for the decreasing Pb concentration in plant tissues (**Figure 3**). Both BC and BCC markedly increased SOC and MBC due to the presence of significant amounts of carbon in BCC and BCC (Jiang et al., 2019; Zheng et al., 2022). Biochar-mediated increase in SOC depends on feedstock type, pyrolysis conditions, application rates, and soil properties (El-Naggar et al., 2019; Yang and Lu, 2020). The increase in MBC and SOC following the addition of different amendments caused a marked increase in plant growth.

The bioaccumulation concentration and bio-concentration factor of *Brassica* plants were significantly increased under Pb stress. Conversely, BC and BCC significantly reduced the bioaccumulation concentration and bio-concentration factor. The lower BAC and BAF in BCC and BC treatment resulted from the lower uptake of Pb stress by plant roots (Ali et al., 2017). BC absorbs HMs, which caused their chelation and reduced their translocation to above-ground plant parts, therefore resulting in less BAC and BAF (Antonangelo and Zhang, 2020). Furthermore, the DIM and HRI values were less than 1 with BC and BCC application, indicating that consumption of *Brassica* was safe.

## Conclusion

Lead stress caused a serious reduction in the growth, yield, and oil contents of *Brassica* by impairing plant physiological and biochemical parameters due to increased Pb uptake. Biochar and biochar co-compost effectively abated the toxicity of lead and markedly improved the *Brassica* seed and oil yields by improving physiological and biochemical functioning and soil properties and restricting the uptake and accumulation of Pb. The application of BCC also enhanced the phyto-stabilization efficiency as evidenced by minimum BAC and BAF, and it also reduced the HR linked with the use of *Brassica* as indicated by the lower values of DIM, HRI, and THQ. This study was conducted in controlled conditions; therefore, future studies must be performed in open-field conditions to assess BCC’s impacts against Pb toxicity. Additionally, studies are also needed to explore the molecular mechanisms mediated by BC to induce Pb tolerance in plants.

## Data Availability

The original contributions presented in the study are included in the article/supplementary material. Further inquiries can be directed to the corresponding authors.
